# Automated image fusion during endovascular aneurysm repair: a feasibility and accuracy study

**DOI:** 10.1007/s11548-023-02832-2

**Published:** 2023-01-31

**Authors:** Stefan P. M. Smorenburg, Rutger J. Lely, Iris Smit-Ockeloen, Kak Khee Yeung, Arjan W. J. Hoksbergen

**Affiliations:** 1 Department of Surgery, Amsterdam University Medical Centers, Vrije Universiteit, Room J1A-222, Postbox 22660, 1100 DD Amsterdam, The Netherlands; 2 Department of Radiology, Amsterdam University Medical Centers, Vrije Universiteit, Amsterdam, The Netherlands; 3 Amsterdam Cardiovascular Sciences, Amsterdam, The Netherlands; 4 Department of Image Guided Therapy, Philips, Best, The Netherlands

**Keywords:** Image fusion, Fusion imaging, 2D-3D, Automated, 3D roadmap, EVAR, Navigation, Overlay

## Abstract

**Purpose:**

Image fusion merges preoperative computed tomography angiography (CTA) with live fluoroscopy during endovascular procedures to function as an overlay 3D roadmap. However, in most current systems, the registration between imaging modalities is performed manually by vertebral column matching which can be subjective, inaccurate and time consuming depending on experience. Our objective was to evaluate feasibility and accuracy of image-based automated 2D-3D image fusion between preoperative CTA and intraoperative fluoroscopy based on vertebral column matching.

**Methods:**

A single-center study with offline procedure data was conducted in 10 consecutive patients which had endovascular aortic repair in which we evaluated unreleased automated fusion software provided by Philips (Best, the Netherlands). Fluoroscopy and digital subtraction angiography images were collected after the procedures and the vertebral column was fused fully automatically. Primary endpoints were feasibility and accuracy of bone alignment (mm). Secondary endpoint was vascular alignment (mm) between the lowest renal artery orifices. Clinical non-inferiority was defined at a mismatch of < 1 mm.

**Results:**

In total, 87 automated measurements and 40 manual measurements were performed on vertebrae T12–L5 in all 10 patients. Manual correction was needed in 3 of the 10 patients due to incomplete visibility of the vertebral edges in the fluoroscopy image. Median difference between automated fusion and manual fusion was 0.1 mm for bone alignment (*p* = 0.94). The vascular alignment was 4.9 mm (0.7–17.5 mm) for manual and 5.5 mm (1.0–14.0 mm) for automated fusion. This did not improve, due to the presence of stiff wires and stent graft.

**Conclusion:**

Automated image fusion was feasible when all vertebral edges were visible. Accuracy was non-inferior to manual image fusion regarding bone alignment. Future developments should focus on intraoperative image-based correction of vascular alignment.

**Supplementary Information:**

The online version contains supplementary material available at 10.1007/s11548-023-02832-2.

## Purpose

Modern hybrid operating rooms have integrated solutions for endovascular aortic procedures combining fixed C-arms with advanced imaging capabilities [1]. A more recent imaging technique is image fusion, which merges preoperative computed tomography angiography (CTA) with live intraoperative fluoroscopy. A patient-specific 3D roadmap is superimposed onto the fluoroscopy images, enabling the operator to insert stent grafts, wires and catheters guided by the 3D roadmap for easier cannulation of aortic side branches [2]. Image fusion was first introduced in 2015. Over the years, utilization resulted in a reduction in radiation exposure and contrast volume in mostly complex but also in standard endovascular aortic aneurysm repair (EVAR) [3–13]. However, during EVAR, the fusion registration between preoperative CTA and live fluoroscopy is performed by manually matching of the vertebral column, making the technique operator-dependent and time consuming depending on experience [14]. Current image fusion systems are designed as hardware-based systems that match the coordinate system of the C-arm and operation table, with preoperative CTA. Due to exponential growth of computer power, it is now possible to automate such tasks and change from hardware-based to image-based registration [15–19]. The aim of this experimental study was to assess the feasibility and accuracy of automated image-based 2D-3D registration, which automatically fuses preoperative CTA and a single-shot intraoperative fluoroscopy, based on vertebral column matching. The hypothesis is that automated image fusion is feasible and accurate. The ultimate goal is to standardize this step during EVAR which will improve the ease of use of image fusion registration for operators.

## Methods

### Study design

**Fig. 1:**
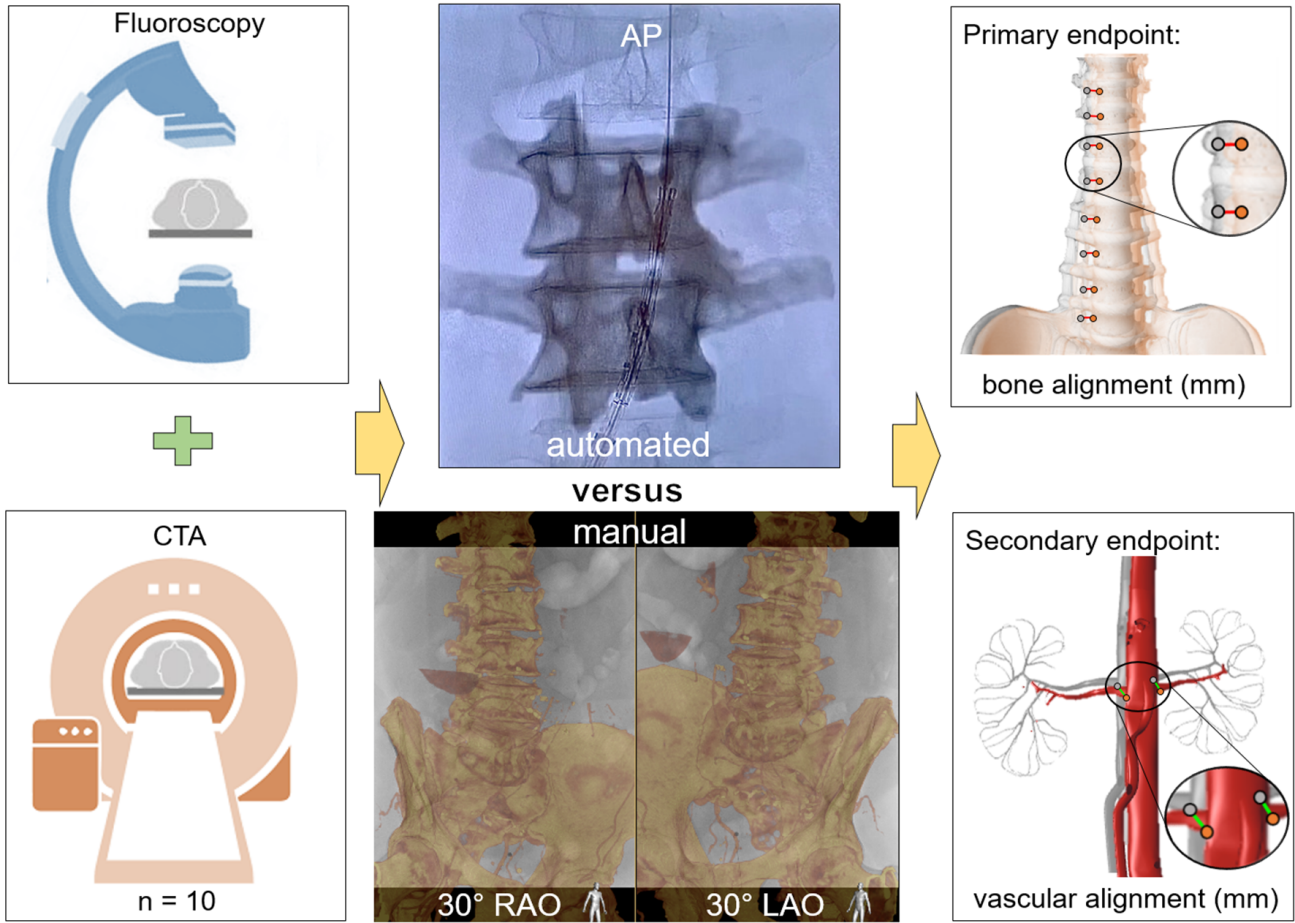
Diagram of the study protocol. First, CTA and fluoroscopy were acquired during an EVAR procedure. After the procedure, the images were retrieved and the vertebral column was fused automatically by the registration algorithm on an anteroposterior (AP) image. After this, regular manual image fusion was performed, 30° right anterior oblique (RAO) and left anterior oblique (LAO), in the identical patients to function as a control group. To correct for inter-observer variability, this was performed by two physicians. Fusion results were assessed on bone alignment measurements (mm) at the lateral edges of the vertebrae and vascular alignment measurements at the lowest renal artery orifice

A single-center, experimental study with retrospective data was conducted of 10 consecutive patients which had undergone EVAR for infrarenal abdominal aortic aneurysm (AAA). The study was approved by the Ethics Committee of Amsterdam University Medical Centers. New automated fusion imaging software was provided by Philips (Best, the Netherlands). Feasibility and accuracy was tested by comparing the automated fusion imaging with conventional manual image fusion (Fig. 1). Feasibility was defined as the ability of the image fusion algorithm to perform the fusion automatically with no need for manual adjustment. Accuracy was defined as bone alignment in millimeters, measured at the edges of the vertebral column, between preoperative CTA and live fluoroscopy.

### Preprocedural CTA protocol

Typically between 1 to 3 months before EVAR, all patients had undergone computed tomography angiography (CTA). CTA imaging was acquired with a slice thickness varying between 0.625 mm and 1 mm (scan settings: 512 × 512 pixels, voxel size: 1 × 1 × 0.9 mm, 120 kV and 130 mAs). Iodine-based contrast was intravenously administered with a volume of 100 mL, 5 mL/s and 300 mg Iodine/mL (UltraVist; Bayer HealthCare AG, Berlin, Germany) by using a patient-specific timing delay for the arterial phase. After scan acquisition, images were send to the hospital PACS system.

### Automated image fusion registration

**Fig. 2:**
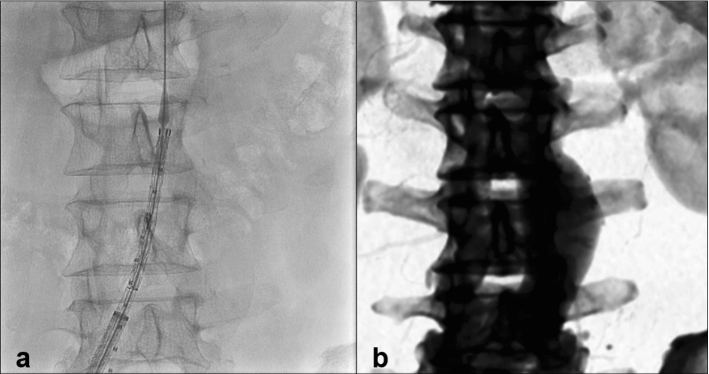
Imaging used for automated fusion: first fluoroscopy image of the initial DSA (**a**) and preoperative CTA converted to a digital X-ray projection (**b**)

From the hospital PACS system, 10 consecutive patients were selected who had undergone standard EVAR for infrarenal AAA after June 1, 2020. A description of the procedure is detailed in Appendix A. The preprocedural CTA and intraoperative initial DSA images, before stent graft deployment, were exported from the hospital PACS archive and used for prospective automated image fusion analysis. The images were uploaded anonymously into new fusion imaging software provided by Philips (Best, the Netherlands) hosted on an online server by Amazon Web Services (Amazon, Seattle, USA) located within the European Union. The first fluoroscopy image of the initial DSA of the EVAR procedure was displayed on the left side of the screen (without subtraction mask, Fig. 2a) and CTA on the right side of the screen (Fig. 2b). The patient’s vertebral column was automatically segmented from the CTA and automatically converted to a digital X-ray projection. The automated registration was manually started and, based on a variable step-size affine projection algorithm, developed in-house by Philips. It rotates, translates and scales the vertebral column from the CTA to the orientation and position of the vertebral column of the fluoroscopy. By doing this, patient position mismatch between CTA and the operating room could be corrected. In each patient, the registration process was executed per vertebrae pair T12–L1, L2–L3 and L4–L5. Since the outer edges of the vertebrae are most distinguished on fluoroscopy, the algorithm identifies these edges. First, a global search was performed, to find the most optimal vertebral pair match between a 30° right anterior oblique (RAO) view and a 30° left anterior oblique (LAO) view to mimic C-arm angulations during an EVAR procedure. Registration scores were calculated in the background and the x-, y- and z-coordinates were displayed. During this process, the user sees the CTA vertebrae pairs moving superimposed onto the fluoroscopy image. After the global search was finished, a visual inspection was performed and the vertebral pair with the highest registration score was selected for local registration. If during visual inspection no realistic match was found, the user adjusted the vertebral column manually to provide a global match. This is marked as ‘manual adjustment needed’ for feasibility assessment. During local registration, the vertebrae pair was translated, rotated and scaled again but with smaller steps to reach the best registration achievable. After the automated vertebral column fusion was finished, the vascular anatomy of the aorta was displayed onto the initial EVAR DSA of the procedure. The DSA frame was selected in which the true lumen of the lowest renal artery orifice was visible to compare with the superimposed renal artery orifice of the preoperative CTA, and calculations were made for potential mismatch.

### Manual image fusion registration as control group

To function as a control group, we performed a secondary assessment of manual image fusion on the same 10 consecutive EVAR patients. To correct for inter-observer variability, two physicians, one with 5+ years of image fusion experience (RL) and one physician with 2 year experience with image fusion (SS), performed manual image fusion registration on the identical patients by recalling the procedural images from the workstation, repeating the manual image fusion registration steps by matching the vertebral column (Appendix A). After manual registration, the initial DSA was recalled and the frame was selected in which the lowest renal artery orifice was best visualized. The manually fused RAO 30° and LAO 30° registration images and the initial DSA fusion images were exported anonymously from the hybrid operating room for further analysis (Fig. 1).

### Primary endpoint

*Feasibility* was defined as the ability of the image fusion algorithm to perform the fusion automatically with no need for manual adjustment. This was documented per patient.

*Accuracy* was defined as bone alignment measurements in millimeters between CTA and fluoroscopy of the edges of the vertebral column. The main accuracy outcome is the median difference between the automated and manual bone alignment measurements.

### Bone alignment

All images of manual image fusion and automated image fusion were uploaded in medical image viewer Horos, version 4.0 (Horos Project, Annapolis, MD, USA).

**Fig. 3:**
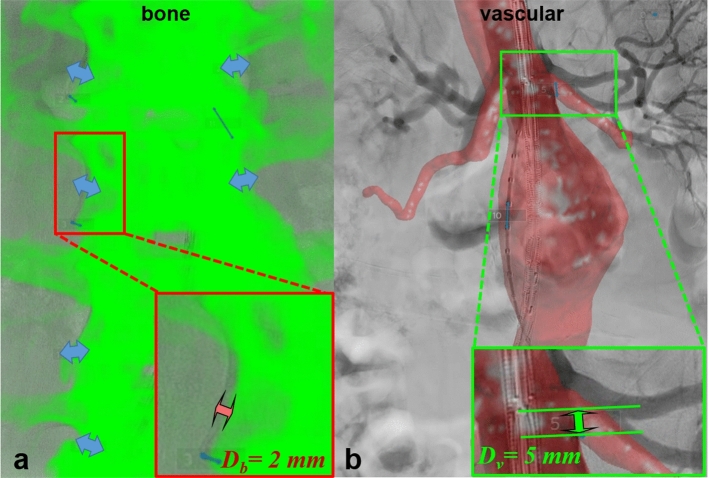
Automated registration evaluation (**a**) by measuring bone alignment in millimeters at the lateral side of each vertebral body (Db) and live guidance evaluation (**b**) by measuring vascular alignment orifice displacement of the lowest renal artery compared with the initial DSA (Dv). Note that for the bone alignment an average is calculated of the two lateral vertebra measurements

For bone alignment, the lateral edges of the patients’ vertebrae were identified by visual inspection. The offset between the identical vertebral edges was measured with a digital measurement tool (caliper) between the CTA and fluoroscopy image (Fig. 3a). The pigtail markers visible each 10 mm functioned as a calibration for the caliper. The bone alignment measurement was performed for both lateral sides of each individual vertebra visible in the image, in one image plane (*x* and *y*-axis), see also Fig. 3a, b. An average was calculated per vertebral pair between the left lateral alignment and right lateral alignment value, given the assumed anatomical symmetry of the individual. The measurement protocol was identical for automated and manual registration. The only difference was that bone alignment measurements were performed in one fluoroscopy image for automated image fusion (anteroposterior) and in two fluoroscopy images for manual image fusion; 30° right anterior oblique (RAO), 30° left anterior oblique (LAO).

### Secondary endpoint

#### Vascular alignment

For vascular alignment, the orifice of the lowest renal artery was identified and the offset between the identical orifice location was measured between preoperative CTA and the initial DSA at the moment of lowest renal artery visibility (Fig. 3b). The most distal edge of the orifice was defined as starting and endpoint.

### Data collection and statistical analysis

Bone alignment and vascular alignment are represented as median with interquartile range (IQR). The Mann–Whitney *U* test was used to test for significant differences in accuracy between manual and automated fusion. Inter-observer agreement between the two physicians in the manual control group was determined with the interclass correlation coefficient (ICC) (agreement, 2-way-mixed, single measure). An ICC of 1.0 equals perfect agreement, ICC > 0.85 equals an excellent agreement, ICC between 0.75–0.85 equals good agreement, ICC between 0.40–0.75 equals fair agreement, and ICC < 0.40 equals poor agreement. The automated image fusion alignment results were compared with the manual image fusion alignment results, and median differences were calculated.

## Results

In total, 87 automated measurements and 40 manual measurements were performed on vertebrae T12–L5 in all 10 patients (Table 1). The median age of the patients (9 men, 1 woman) was 73.0 years (IQR 70.0–73.0), median body mass index was 25.3 kg/m^2^ (IQR 22.1–30.2), and median aneurysm diameter was 61.0 mm (IQR 55–65). All stent grafts implanted were Cook Zenith Alpha (Cook Medical, Bloomington, Ind). Median dose area product (DAP) of all procedures was 148 Gy-cm^2^ (IQR 120–170), and median fluoroscopy time was 19.5 min (IQR 18.5–46).

**Table 1:** Automated registration and alignment measurement results for 10 patients

Patient	Registration	Vertebrae	*M*_bone_ (mm)	*M*_vas_ (mm)
1	Global	L3 L2	2.0	17.0
	Local	L5 L4	0.5	11.0
2	Global	L5 L4	3.0	7.0
	Local	L3 L2	0.0	5.0
3	Global	L5 L4	1.8	16.0
	Local	L5 L4	0.0	13.0
4	Global	L5 L4	2.5	5.0
	Local	L5 L4	0.0	4.0
5	Global*	L3 L2	1.5	9.0
	Local	L3 L2	1.5	9.0
6	Global	L1 T12	2.5	3.0
	Local	L3 L2	0.5	2.0
7	Global	L3 L2	1.3	1.0
	Local	L5 L4	1.0	1.0
8	Global*	L1 T12	0.0	4.0
	Local	L1 T12	0.0	4.0
9	Global	L5 L4	0.8	11.0
	Local	L3 L2	0.0	6.0
10	Global*	L1 T12	1.0	17.0
	Local	L5 L4	1.0	14.0
*Median global*			**1.6**	**8.0**
	min/max		(0–3.0)	(1.0–17.0)
*Median local*			**0.3**	**5.5**
	min/max		(0–1.0)	(1.0–14.0)

Total median results are highlighted in bold

Displaying registration type (first a global registration is performed followed by a local registration), vertebrae pair, bone alignment (*M*_bone_) and vascular alignment (*M*_vas_)

*Manual adjustment needed

### Primary endpoints

#### Feasibility

Fully automated image fusion was feasible in 7 out of 10 patients. Manual adjustment was needed in 3 of the 10 patients. The reason for this was that in these cases, the vertebral edges were not completely visible on the fluoroscopy image. Since this was real-world acquired procedural data, some initial angiograms were performed with the vertebrae on the outside border of the screen. When a segment of the vertebra was not visible, the registration algorithm was not able to perform the registration task fully automatically, and manual adjustment was needed.

#### Automated fusion and bone alignment measurement

The registration algorithm automatically registered the predefined 3 vertebrae pairs (T12–L1, L2–L3, L4–L5) for each patient. The registration algorithm first needed an initial estimate of the bone registration, which is referred to as the global phase. After this, the registration algorithm can perform a second registration, which is referred to as the local phase. During the global registrations, vertebrae pair T12–L1 was selected three times, L2–L3 three times and L4–L5 four times. During the subsequent local registration phase, vertebrae pair L1–T12 was selected one time, L2–L3 four times and L4–L5 five times. The translation parameters varied between minimum and maximum values of *T*_*x*_: − 15.15 and 55.08, *T*_*y*_: − 140.39 and 107.28 and *T*_*z*_:− 227.19 and 49.54. The rotation parameters varied between minimum and maximum values of *R*_*x*_:− 39.62 and 9.0, *R*_*y*_: − 29.71 and 10.78 and *R*_*z*_: − 1.16 and 6.51.

Bone alignment measurements *M*_bone_ resulted in a median global offset of 1.6 mm (min/max: 0;3.0) and median local offset of 0.3 mm (min/max, 0;1.0). Note these values are solely the bone alignment measurement (no comparison with the control group), and values are displayed in Table 1.

#### Manual fusion and alignment measurements: control group

For the manual control group, median bone alignment for physician 1 was 0.2 mm (min/max, 0;1.0) and for physician 2 was 0.3 mm (min/max, 0.1;1.0) (Table 2). Since the ICC was 0.66 (*p* = 0.074, fair agreement) for bone alignment, the physician results were averaged which resulted in a total manual bone offset median of 0.2 mm (min/max, 0.1–0.9).

**Table 2:** Manual fusion and alignment measurements (mm) (n = 10) performed by two physicians

Pat	Bony alignment		Total	Vascular alignment		Total
	Physician 1	Physician 2		Physician 1	Physician 2	
1	0.7	1.0	0.9	8.3	7.3	7.8
2	0	0.3	0.2	4.2	3.9	4.1
3	0.3	0.5	0.4	18.0	17.0	17.5
4	0.9	0.2	0.1	9.9	8.3	9.1
5	0.0	0.3	0.2	14.0	15.0	14.5
6	0.3	0.1	0.2	5.6	5.6	5.6
7	0.5	0.1	0.3	3.3	0.6	2.0
8	0.1	0.2	0.2	2.0	2.1	2.1
9	0.1	0.3	0.2	0.5	0.9	0.7
10	1.0	0.8	0.9	2.2	2.5	2.4
Median	0.2	0.3	0.2	4.9	4.8	4.9
min/max	(0–1.0)	(0.1–1.0)	(0.1–0.9)	(0.5–18.0)	(0.6–17.0)	(0.7–17.5)
ICC*	0.66 (*p* = .074)		–	0.98 (*p* = .00)		–

From the results of both physicians, the medians were calculated and range (min/max)

^*^ICC = intraclass correlation coefficient

#### Main comparison: automated versus manual fusion

The median difference was calculated between automated and manual image fusion bone alignment measurements. (Table 3). These results were used as main alignment outcomes. For bone alignment, this resulted in a total median difference of 0.1 mm (*p* = 0.94).

**Table 3:** Median difference (mm) between automated and manual image fusion

		Total	min/max	Median difference	*p* value*
Bone alignment	Manual	0.2	(0.1–0.9)	0.1	0.94
	Automated	0.3	(0–1.0)		
Vascular alignment	Manual	4.9	(0.7–17.5)	0.6	0.68
	Automated	5.5	(1.0–14.0)		

^*^Mann–Whitney *U*

### Secondary endpoints

#### Vascular alignment

For automated image fusion, vascular alignment measurements *M*_vas_ resulted in a median global vascular alignment of 8.0 mm (min/max: 1.0;17.0) and median local vascular alignment of 5.5 mm (min/max, 1.0;14.0) (Table 1).

For manual image fusion, the median vascular alignment for physician 1 was 4.9 mm (min/max, 0.5;18.0) and physician 2 was 4.8 mm (min/max, 0.7;17.5). The ICC resulted in 0.99 (*p* = 0.00, excellent agreement), and after averaging the measurements of physician one and two, the median physician vascular offset was 4.9 mm (0.7–17.5) (Table 2).

The median difference between automated and manual image fusion resulted in a total median difference of 0.6 mm (*p* = 0.68) (Table 3).

#### Registration time

Automated registration time was 14.5 min (global) and 20 s (local) for the first 9 patients resulting in 15 min. For the last patient, this improved to 2 min (global) and 10 s (local) resulting in 2.2 min, due to updated server capacity during the registration process.

## Discussion

In this study, we have demonstrated that automated image fusion with real-world EVAR data is feasible and accurate, as compared to conventional manual image fusion. The measured bone alignment differs 0.1 mm between the automated and manual registration process, reaching the predefined clinical non-inferiority.

This is important as current image fusion applications require a significant amount of human interaction. By automating the image fusion registration step during EVAR, the system can perform this task in the background and eventually standardize this step in the process. More importantly, image-based registration instead of hardware-based registration can result in a more accurate vascular overlay which is the ultimate goal of precise image fusion during EVAR procedures.

Previous studies have also evaluated automated image fusion for CTA and fluoroscopy. Varnavas and Carrel et al. demonstrated in 2015 an automated 2D–3D algorithm which was able to perform fully automated image fusion [14]. More recently, Rolls and Maurel et al. demonstrated the utilization of fully automated 2D-3D image fusion with Cydar EV system (Cydar Medical, Cambridge, UK) [19]. Bone alignment measurements were not presented; however, after a comparison with hardware-based image fusion, the image-based image fusion system was superior. Kaladji et al. reported a similar image-based fusion system, EndoNaut (Therenva, Rennes, France) [20]. Bone alignment values were not presented; however, automated image fusion was demonstrated to function correctly. The aforementioned authors also highlighted the benefits of automated image fusion, removing the cumbersome user interactions, and the possibility of standardization of the registration steps and a faster and smoother workflow for the physician.

The fusion of a preoperative modality such as CTA with live procedural fluoroscopy images is inherently sensitive for numerous sources of error as also described in several reports [21–26]. First, vascular deformation occurs in the aorta and iliac arteries after insertion of stiff guidewires and stent grafts. This was also present during our study since we measured the vascular alignment during initial DSA, after stiff endovascular devices were already introduced. These results are displayed in Table 2 (per patient) and Table 3 (mean values). Second, patient motion during draping and device manipulations is strongly correlated with displacement of the lowest renal artery. Third, EVAR is mostly performed with the arms-down position, whereas CT is generally acquired with the patient in arms-up position. Subsequently, endorotation and exorotation of patient’s legs may result in pelvic bone mismatch between CT and fluoroscopy when performing manual image fusion. This is why manual image fusion emphasis should be the vertebral column only.

The primary limitation to the generalization of the study results is the use of retrospective data. In all EVAR procedures, the first angiography was done with the stiff guidewire and stent graft already introduced, causing vascular mismatch due to distortion of the aortoiliac arteries. To assess the true vascular accuracy of automated image fusion, an angiography should be performed without any stiff device introduced. Furthermore, clinical data of only 10 patients was analyzed, which is a limited cohort. Additionally, the time to perform automated registration was unsatisfactory (14 min). This was improved by enhancing the computing power in patient 10, resulting in a much faster (2 min) automated registration time. The inter-observer variability between both physicians performing manual image fusion was fair, which is satisfactory but can be improved. This also demonstrates the added value of standardization of the registration step during image fusion.

We have demonstrated that automated image fusion bone registration is feasible and accurate, if the vertebrae were completely visible. If the physician is made aware that the vertebrae during a single-shot fluoroscopy at the start of the EVAR procedure should be visualized completely, the algorithm can perform automated image fusion in the background. Future research should focus on the development of several features of automated image fusion but mainly on automated correcting for vascular deformity for the visceral and iliac arteries. This could be performed by integrating an iterative process; when the guidewire and stent graft are detected and move outside the image fusion overlay, it will automatically deform the image fusion overlay to match the already introduced guidewire and stent graft [26]. Furthermore, cloud-based strategies to implement these techniques offer the ability to improve fusion imaging registration with artificial intelligence techniques [27].

## Conclusion

Automated image fusion registration is feasible for endovascular aortic repair and accuracy was non-inferior to manual image fusion. To minimize user interactions, future developments should focus on the automated image-based correction of vascular alignment.

## Supplementary Information

Below is the link to the electronic supplementary material.

Supplementary file1 (DOCX 14 kb)
