# Supplementary material for: Automated image fusion during endovascular aneurysm repair: a feasibility and accuracy study
Source: Int J Comput Assist Radiol Surg. 2023 Jan 31;18(8):1533–41. doi: 10.1007/s11548-023-02832-2 (PMC10363050; doi:10.1007/s11548-023-02832-2)
Supplement: Supplementary file 1 — Supplementary file1 (DOCX 14 kb) [file 11548_2023_2832_MOESM1_ESM.docx]

# Appendix a. Supplementary data

## EVAR procedure with image fusion

All patients were operated on the hybrid operating theatre and fluoroscopy imaging was acquired with a ceiling-mounted C-arm (Azurion Flexmove 7 C20, Philips, Best, the Netherlands). Ultrasound guided percutaneous or cut down access was gained in both femoral arteries. The C-arm X-ray system was moved into position of the abdominal area of the patient. Manual image fusion was performed by acquiring two 2D fluoroscopy images which were acquired with an angular difference >60° in the right and left anterior oblique plane (RAO / LAO), typically RAO 30° and LAO 30°, or at 45°. After the two fluoroscopy images were obtained, manual registration (2D-3D) was performed by aligning the vertebral column of the superimposed CTA with the vertebral column of the fluoroscopy by rotating and translating the images manually. Emphasis was placed on vertebrae T12/L1 as this is the area where the renal arteries branch off and the stent graft was deployed. After visual inspection by the operator, live guidance was selected and the aortoiliac arteries were superimposed onto the fluoroscopy image, facilitating guidewire and stent graft navigation. The main body of the stent graft was introduced through the anatomically most suitable iliac side and positioned at the renal arteries. Digital subtraction angiography (DSA) was performed to visualize the lowest renal artery orifice during patient breath hold at 3 frames per second, low-dose abdomen protocol, with the power injector set at 20 mL Iodine at 15 mL/s, (300 mg Iodine/mL). When the image fusion overlay was not perfectly matched with the renal arteries, the overlay was moved manually (only in-plane) to match the true renal artery orifices. The stent graft was deployed below the renal arteries, and completed with contralateral and ipsilateral legs in a standard fashion after which a final DSA was performed to check for endoleaks, stent graft position and patency of renal and iliac arteries.
